# Modifications in Lipid Levels Are Independent of Serum TNF-α in Rheumatoid Arthritis: Results of an Observational 24-Week Cohort Study Comparing Patients Receiving Etanercept Plus Methotrexate or Methotrexate as Monotherapy

**DOI:** 10.1155/2014/510305

**Published:** 2014-08-27

**Authors:** Norma Alejandra Rodriguez-Jimenez, Carlos E. Garcia-Gonzalez, Karina Patricia Ayala-Lopez, Benjamin Trujillo-Hernandez, Erika Anita Aguilar-Chavez, Alberto Daniel Rocha-Muñoz, Jose Clemente Vasquez-Jimenez, Eva Olivas-Flores, Mario Salazar-Paramo, Esther Guadalupe Corona-Sanchez, Monica Vazquez-Del Mercado, Evangelina Varon-Villalpando, Adolfo Cota-Sanchez, Ernesto German Cardona-Muñoz, Jorge I. Gamez-Nava, Laura Gonzalez-Lopez

**Affiliations:** ^1^Programa de Doctorado en Farmacologia, Centro Universitario de Ciencias de la Salud (CUCS), Universidad de Guadalajara (U de G), Guadalajara, JAL, Mexico; ^2^Hospital de Especialidades, Centro Medico Nacional de Occidente, Instituto Mexicano del Seguro Social (IMSS), Guadalajara, JAL, Mexico; ^3^Departamento de Anestesiología, Hospital Regional de Zona 1, IMSS, Aguascalientes, AGS, Mexico; ^4^Centro Universitario de Investigaciones Biomédicas, Universidad de Colima, Colima, COL, Mexico; ^5^Programa de Becarios en Investigacion en Salud, Unidad de Medicina Familiar 2, IMSS, Guadalajara, JAL, Mexico; ^6^Departamento de Anestesiología, Hospital General Regional 180, IMSS, Tlajomulco, JAL, Mexico; ^7^Division de Investigacion en Salud, UMAE, Hospital de Especialidades, Centro Medico Nacional de Occidente, IMSS, Mexico; ^8^Departamento de Fisiologia, CUCS, U de G, Guadalajara, JAL, Mexico; ^9^Coordinacion de Posgrado, CUCS, U de G, Guadalajara, JAL, Mexico; ^10^Departamento de Patologia Clinica, Hospital General Regional 110, IMSS, Guadalajara, JAL, Mexico; ^11^Unidad de Investigacion en Epidemiologia Clinica, Hospital de Especialidades, Centro Medico Nacional de Occidente, IMSS, Guadalajara, JAL, Mexico; ^12^Departamento de Medicina Interna-Reumatologia, Hospital General Regional 110, IMSS, 44716 Guadalajara, JAL, Mexico; ^13^Programa de Doctorado en Salud Publica, CUCS, U de G, 44340 Guadalajara, JAL, Mexico

## Abstract

*Objective.* To compare the modifications in lipids between patients with rheumatoid arthritis (RA) receiving etanercept plus methotrexate (ETA + MTX) versus methotrexate (MTX) and their relationship with serum levels of tumor necrosis factor-alpha (TNF-α). *Methods.* In an observational cohort study, we compared changes in lipid levels in patients receiving ETA + MTX versus MTX in RA. These groups were assessed at baseline and at 4 and 24 weeks, measuring clinical outcomes, total cholesterol, triglycerides, high-density lipoprotein cholesterol (HDL-C), low-density lipoprotein cholesterol, and TNF-α. *Results.* Baseline values for lipid levels were similar in both groups. HDL-C levels increased significantly only in the ETA + MTX group (from 45.5 to 50.0 mg/dL at 4 weeks, a 10.2% increase, *P* < 0.001, and to 56.0 mg/dL at 24 weeks, a 25.1% increase, *P* < 0.001), while other lipids underwent no significant changes. ETA + MTX also exhibited a significant increase in TNF-α (44.8 pg/mL at baseline versus 281.4 pg/mL at 24 weeks, *P* < 0.001). The MTX group had no significant changes in lipids or TNF-α. Significant differences in HDL-C between groups were observed at 24 weeks (*P* = 0.04) and also in TNF-α  (*P* = 0.01). *Conclusion.* HDL-C levels increased significantly following treatment with ETA + MTX, without a relationship with decrease of TNF-α.

## 1. Introduction 

Cardiovascular disease constitutes the main cause of death for patients with long disease duration in rheumatoid arthritis (RA) [1]. A recent study observed that patients with RA have at least a 1.6-fold increased risk for acute myocardial infarction and ischemic stroke compared with controls [2], whereas the frequency of dyslipidemia in RA may range from 28 to 49% [3, 4]. Dyslipidemia is influenced by a multiplicity of factors, including activity and disease duration, comorbidity, and pharmacological therapies, particularly with corticosteroids [5]. Although, some differences are reported across the results of studies evaluating the lipid profiles in RA, some researchers have observed an increase in the levels of low-density lipoprotein cholesterol (LDL-C) in patients with RA compared with controls [6]; other groups have failed to identify these differences [7]. However, yet other studies have observed lower levels of high-density lipoprotein cholesterol (HDL-C) in patients with RA [6–8]. Currently low levels of HDL-C are considered an independent risk factor for the development of cardiovascular disease [9]. Therefore, some authors consider improvement of levels of this lipoprotein as an outcome measure within the therapeutic goals of dyslipidemia treatments [10].

Tumor necrosis factor-alpha (TNF-α) exerts a multiplicity of effects that are not only related to disease activity. This cytokine also participates in increasing cardiovascular risk factors, including hepatic synthesis of C-reactive protein (CRP) and decreasing of HDL-C levels [11]. Whether or not the blocking of proinflammatory effects induced by TNF-α due to anti-TNF agents in RA would offer benefits in modifying the abnormal lipid profiles should be considered. Etanercept (ETA) is a dimeric fusion protein consisting of two extracellular domains of the human p75 TNF receptor linked with the Fc portion of a type 1 human immunoglobulin. Relevantly, this anti-TNF agent blocks not only TNF-α but also lymphotoxin-α, a cytokine that exerts proatherogenic properties in animal models [12]. However, it remains unknown whether ETA exerts significant clinical effects on the lipid profile when compared with methotrexate (MTX). A systematic review of 24 observational studies evaluated changes in lipid profile in patients with RA treated with diverse anti-TNF agents [13]. This review included only six studies of patients treated with ETA, and these results were mixed with results from patients treated with other anti-TNF agents [13]. Therefore, this review reported wide variability in lipid profile changes following ETA therapy; these effects cannot be attributed to a particular anti-TNF-α agent. In an interesting work, Jamnitski et al. assessed changes in lipid profile in patients with RA receiving ETA as unique anti-TNF agent, although this study was performed without a comparison group; therefore, the effects of potential confounders cannot be excluded [14].

Thus, a lack of comparative studies evaluating the effects of ETA on the lipid profiles of patients with RA renders it uncertain whether modifications in these lipids (if they exist) are related to changes in TNF-α serum levels induced by this anti-TNF agent. Thus, we performed a comparative study evaluating the modifications in lipid levels in patients with RA treated with ETA plus MTX versus patients receiving MTX as monotherapy and elucidating when these changes are related or not with modifications in serum levels of TNF-α.

## 2. Patients and Methods

### 2.1. Study Design

This 6-month prospective cohort study included consecutive patients with RA from an outpatient rheumatology clinic at a secondary-care hospital (Hospital General Regional 110, of the Instituto Mexicano del Seguro Social) in Guadalajara, Mexico. Patients were included if they were adults (≥18 years of age), met 1987 American College of Rheumatology (ACR) criteria for RA, had an active disease defined by disease activity score (DAS 28) > 3.2, and had not received treatment for least 3 months with synthetic disease-modifying antirheumatic drugs (DMARD) or therapy with biological agents. Patients were excluded for the following reasons: pregnancy, treatment with immunosuppressive drug such as cyclophosphamide or azathioprine, receiving prednisone or the equivalent at doses of >10 mg/day, having active infections at the time of study inclusion, receiving antimalarial drugs, and receiving other biological agents or hypolipidemic drugs (statins or fibrates) in the 3 months prior to entry into the study or during the study. Also excluded were patients with comorbidity associated with abnormalities in the lipid profile (such as hypothyroidism, diabetes mellitus, chronic renal failure, nephrotic syndrome, or hepatopathy). We did not exclude smokers or patients with metabolic syndrome (except if they had diabetes mellitus).

### 2.2. Criteria for Treatment Arm Assignment

According to the Mexican guidelines of the treatment for RA used in our hospital, the patients may receive as first option MTX as monotherapy or may initiate a combined therapy with MTX plus a biological agent (usually an anti-TNF agent) specially if the patient is considered with inadequate therapeutic response after at least 3 months of monotherapy with MTX or another DMARD [15]. Therefore, the rheumatologists in the clinical visit decided to institute a therapeutic strategy independently of this study. The strategies that the rheumatologists decided to initiate were one of the following treatments: ETA + MTX (first group): these patients received ETA 25 mg subcutaneously twice weekly plus MTX 10 to 15 mg per week administered orally for 24 weeks, or MTX (second group), as monotherapy, received this drug at a dosage of 10–15 mg weekly administered orally for 24 weeks. All of the dosages of these drugs remained stable throughout the study.

### 2.3. Clinical Assessment and Followup

A structured questionnaire was administered to the patients to evaluate demographic and clinical variables including disease duration, smoking, and comorbidities. Patients were assessed by the same trained researcher at baseline (time of initial prescription) and at 4 and 24 weeks for the following variables: (a) disease activity according to disease activity score (DAS 28), (b) functioning according to Health Assessment Quest-Disability Index (HAQ-DI), and (c) visual analogue scales (VAS, 0–100 mm) for morning stiffness, pain severity, and disease activity assessed by the physician.

During the study, patients received oral nonsteroidal anti-inflammatory drugs (NSAID) and acetaminophen or, if required, diclofenac intramuscularly as adjuvant treatment for joint pain and inflammation. We recommended to all patients the regular practice of exercise and a diet low in fat.

### 2.4. Lipid Profiling and Serum TNF-α Level Measurement

A venous blood sample was obtained at baseline, at 4 and 24 weeks, to determine levels of total cholesterol, triglycerides, HDL-C, and LDL-C. These lipid levels were assessed with commercial kits using the VITROS-800 machine. All measurements were performed at our hospital's central laboratory. Normal reference values are the following: total cholesterol < 240 mg/dL, triglycerides < 160 mg/dL, HDL-C > 35 mg/dL, and LDL-C < 150 mg/dL. A patient was considered with dyslipidemia when the values of total cholesterol, triglycerides, or LDL-C were above normal values or when HDL-C levels were below reference values.

Also at baseline, 4 weeks, and 24 weeks, a second blood sample was taken and serum was obtained in order to measure TNF-α levels. These levels were quantified by enzyme-linked immunoassay (ELISA) using a commercial kit (Quantikine).

Rheumatoid factor (RF) titers and CRP were measured at all visits by nephelometry.

### 2.5. Statistical Analysis

Quantitative variables are expressed as means ± standard deviations (SD), and qualitative variables are expressed as numbers and percentages. Unpaired Student's *t*-tests were utilized for baseline comparisons of quantitative variables between groups (ETA + MTX versus MTX as monotherapy) and chi-square tests (or Fisher exact tests if required) were employed for comparisons of qualitative variables between groups. Correlations between lipid levels and disease activity, HAQ-DI, TNF-α serum levels, CRP concentrations, and RF titers were computed using Pearson correlation coefficients (*r*). Paired Student's *t*-tests were utilized for within-group comparisons of absolute changes in lipid levels, TNF-α levels, and clinical characteristics expressed as quantitative variables at 4 and 24 weeks relative to baseline. Unpaired Student's *t*-tests were performed for comparisons of differences in the relative change of lipid levels and serum TNF-α levels at 4 weeks and 24 weeks between ETA + MTX and MTX as monotherapy groups. Significance was set at a *P* value of ≤ 0.05. All analyses were performed with SPSS ver. 8.0 statistical software.

## 3. Results

Thirty-five patients were included in the study, 22 of whom received ETA + MTX and 13 MTX as monotherapy. There were no statistical differences in the majority of the variables at baseline, including gender, body mass index (BMI), proportion of smokers, or comorbidity (Table 1). Age was significantly higher in the ETA + MTX group than in the MTX group (47.4 ± 8.3 years versus 40.6 ± 10.8 years, resp.; *P* = 0.04). There was a trend toward longer disease duration in ETA + MTX patients versus MTX patients, although this difference did not reach statistical significance (12.3 ± 6.7 years versus 8.2 ± 6.0 years, resp.; *P* = 0.08). Other clinical characteristics including the DAS 28 score, the HAQ-DI score, morning stiffness, pain severity, and disease activity as assessed by the physician did not differ between groups at baseline (Table 1). There were no significant baseline differences between the two groups in lipid levels, CRP levels, and RF titers. Frequency of dyslipidemia at baseline in the ETA + MTX group was similar to that of the MTX group (59 versus 54%, resp.; *P* = 0.76) (Table 1). There was a trend toward higher baseline TNF-α levels in patients receiving ETA + MTX compared with patients receiving MTX as monotherapy, although this difference did not reach statistical significance (129.14 ± 207.70 pg/mL versus 42.36 ± 23.46 pg/mL resp.; *P* = 0.08).

Table 2 depicts the results of the Pearson correlations at baseline among TNF-α levels, clinical variables, lipid levels, RF titers, and CRP concentrations, employing the total pool of patients (*n* = 35). Although a trend was observed between higher TNF-α levels and severity of disease activity as assessed by the physician, this correlation did not achieve statistical significance (*r* = 0.304, *P* = 0.07). TNF-α serum levels did not correlate with clinical variables, RF titers, or levels of CRP, total cholesterol, triglycerides, HDL-C, or LDL-C.

We carried out within-group comparisons between baseline values and measurements taken at 4 and 24 weeks in selected clinical variables, CRP levels, and RF levels (Table 3). The ETA + MTX group experienced significant improvement at 4 and 24 weeks versus baseline in DAS-28 score (*P* < 0.001), HAQ-DI score (*P* < 0.001), and disease activity as assessed by the physician (*P* < 0.001). The group receiving MTX as monotherapy also significantly improved in DAS-28 score at 4 (*P* = 0.01) and 24 weeks (*P* < 0.001), HAQ-DI at 4 (*P* < 0.001) and 24 weeks (*P* < 0.001), and VAS for disease activity at 4 (*P* = 0.001) and 24 weeks (*P* = 0.001). No significant differences were detected in RF titers at 4 and 24 weeks in the ETA + MTX group, whereas the group receiving MTX as monotherapy exhibited a significant decrease in RF titers. In data not shown in the table, none of the patients who were smokers at baseline stopped smoking or decreased their smoking habit.

Treatment effects on lipid profile and TNF-α levels were determined for both groups (Table 4). A within-group comparison of ETA + MTX patients revealed a significant increase in HDL-C levels at 4 (*P* = 0.02) and at 24 weeks (*P* = 0.009) compared with baseline (Table 4). Remarkably, we observed an increase in TNF-α levels in this group at 4 (*P* = 0.03) and at 24 weeks (*P* = 0.007) compared with baseline, while we detected no significant changes in lipid levels or TNF-α levels in the group receiving MTX as monotherapy.

Regarding differences in relative change between the two groups, the MTX as monotherapy group had a significant increase in total cholesterol levels compared with the ETA + MTX group at 24 weeks (*P* = 0.04) (Table 4), while the ETA + MTX group had significantly higher HDL-C levels than the MTX group at 24 weeks (*P* = 0.04) (Table 4). We detected no other statistically significant changes in lipid levels between the two treatment groups. The ETA + MTX group exhibited significantly higher TNF-α levels than MTX group at 4 (*P* = 0.02) and at 24 weeks (*P* = 0.01) (Table 4).

## 4. Discussion 

The results of the present study show that utilization of the ETA + MTX combined therapy significantly increased HDL-C levels with respect to baseline in patients with RA, although this effect was not associated with significant improvement in other lipids. Patients receiving MTX as monotherapy exhibited no significant changes in these lipid levels. Additionally, improvement in HDL-C levels in patients treated with ETA + MTX was not related to decreases in serum TNF-α levels.

Wide variability in the frequency of dyslipidemia has been observed in RA ranging from 28 to 49% of patients depending on the study [3, 4]. We observed abnormalities in lipid levels in more than one-half of our patients, an observation that is in agreement with previous reports that this prevalence increases during episodes of disease activity [16, 17]. A major consequence of dyslipidemia is the development of atherosclerosis and its complications. In this context, Roman et al. reported that 44% of their patients with RA displayed atherosclerotic plaques on carotids compared with only 15% of controls [18]. Additionally, Gonzalez-Juanatey et al. identified an increase in subclinical atherosclerosis assessed by an increase in carotid intima-media wall thickness and carotid plaques in patients with RA, particularly in those with longer disease duration and more extra-articular manifestations [19].

It has been suggested that only a small subset of patients with RA with dyslipidemia are adequately treated with hypolipemic therapy. Maradit-Kremers et al. reported that only 7–15% of their patients with RA and dyslipidemia received hypolipemic treatment in their cohort [4]. Lipid profiles in RA may be affected by several variables, such as comorbidities and drugs used for treatment. Corticosteroids may contribute to dyslipidemia and cardiovascular complications [20], while antimalarials may improve lipid profiles [21]. However, the effects of other antirheumatic drugs, such as MTX, on lipid levels are not consistent across the different studies. In one study Georgiadis et al. observed a significant increase after 1 year in total cholesterol and HDL-C levels in patients with early RA who were treated with MTX at stable doses [22]. However, that study was limited by the absence of a comparison group, and the effects of potential confounding factors cannot be excluded.

Several studies have evaluated changes in lipids levels associated with the use of anti-TNF-α agents, including infliximab, adalimumab, and ETA. However, contradictions in the observations complicate their interpretation. Popa et al. [23] and Tam et al. [24] reported significant modifications in levels of total cholesterol and triglycerides in patients with RA receiving infliximab, but Bosello et al. [25] and Peters et al. [26] did not find significant changes in lipid levels after treatment with infliximab. Studies using adalimumab have also shown contradictory results [23, 27, 28]. Popa et al. [23] and Gonzalez-Juanatey et al. [27] identified an improvement in levels of total cholesterol and HDL-C in patients with RA treated with adalimumab but the lipid profiles reported by Wijbrandts et al. [28] did not changed significantly with this anti-TNF agent. Anti-TNF agents also have shown effects on endothelial function. Gonzalez-Juanatey et al. observed the effects of adalimumab on endothelial function in patients with RA refractory to DMARD. These authors observed a significant increase in flow-mediated endothelium-dependent vasodilation after 2 weeks and 12 months of adalimumab therapy [29]. However, these authors failed to find significant differences in lipid levels and atherogenic index with treatment with adalimumab [29]. These data underscore the fact that anti-TNF alpha benefits on cardiovascular diseases are produced by multiple mechanisms independently of modifications in the lipid profile.

Although eight studies have assessed the modifications in lipid profiles in patients with RA using ETA [14, 30–36], the results are controversial. Jamnitski et al. observed a significant increase in total cholesterol and triglycerides after 1 year of treatment with ETA [14], whereas Garcês Da Gama et al. observed, in a mixture group compounded by RA patients, ankylosing spondylitis patients, and psoriatic arthritis patients, an increase in HDL-C concentration after the administration of ETA [30], and Seriolo et al. observed, in independent studies, a significant increase in total cholesterol and HDL-C [31–33]. On the other hand, three independent studies performed by del Porto et al., Soubrier et al., and Senel et al. did not observe a change in lipid values in their patients treated with ETA [34–36]. The majority of these studies are limited in their ability to attribute their results to this anti-TNF agent due to the presence of potential confounders. Nearly all of these investigations included patients treated with other anti-TNF-α agents; therefore, the statistical analyses performed combined the results to detect the possible modifications of anti-TNF-α agents as a group, rather than seeking to identify specific drug effects [32–34]. From these studies, Garcês Da Gama et al. [30] separately analyzed patients treated with ETA and treated with infliximab, although they mixed in their results with those of patients with RA, psoriatic arthritis, and ankylosing spondylitis. In the study of Jamnitski et al. [14] are evaluated the benefits on the lipid profile in a cohort of patients with RA treated with ETA, but this study lacked a comparison group and did not separately analyze the results in patients treated exclusively with ETA and patients treated with ETA + DMARD combination. Given that some DMARD, such as antimalarials, may improve lipid levels [21], considering the effects of DMARD on changes in lipid profile is required. Therefore, we evaluated the effects of ETA + MTX versus MTX alone on lipid profile without including other anti-TNF-α agents or other DMARD except for MTX. Additionally, it is relevant to consider that from eight investigations performed with the aim to evaluate the effects of ETA on the lipid profile in RA, only three [32–34] utilized a comparison group, with this group being a requirement for evaluating the magnitude effect. Therefore, in order to assess effectively whether the effect of ETA + MTX on lipid levels is relevant, we included, as comparison group, patients receiving treatment with MTX as monotherapy. Interestingly, some studies have reported the effects of other biological agents different from anti-TNF inhibitors in patients with RA refractory to anti-TNF agents. In this context, rituximab has shown a short-term increase of LDL-C and total cholesterol when compared with base line values. However, these early differences observed at week 2 did not remain at month 6 [37]. On the other hand, the effect of interleukin-6 (IL-6) receptor blockade with tocilizumab has been associated with an increase of total cholesterol, LDL-C, and HDL-C [38].

The positive effects of anti-TNF agents entertain multiple advantages in lipid profiles of patients with RA. In RA, the frequency of metabolic syndrome is around 33% [39] with no previous events of cardiovascular disease. Some adipokines participate in the pathogenesis of atherosclerosis and are also mediators of inflammation; among these, leptin and adiponectin are highly relevant in RA. Leptin is a proinflammatory cytokine produced by adipocytes, with these being potent stimuli for its production of some cytokines such as TNF-α and IL-1*β*. This adipokine exerts several effects on atherosclerosis, such as inducing endothelial dysfunction, increasing oxidative stress, increasing platelet aggregation, and stimulating the migration, proliferation, and hypertrophy of smooth muscle cells on the vascular wall [40]. Adiponectin has, instead, some opposite effects on leptin; adiponectin possesses anti-inflammatory properties, and also high levels are associated with antiatherogenic effects, increasing insulin sensitivity. Low adiponectin levels are associated with the presence of metabolic syndrome, dyslipidemia, and higher plasma glucose, contributing to the development of atherosclerosis [40]. Gonzalez-Gay et al. describe an association between CRP levels and adiponectin concentrations [41]. These adiponectin circulating concentrations also correlated negatively with triglyceride/HDL-C ratios, total cholesterol/HDL-C ratios, and high fasting plasma glucose levels, independent of CRP levels and BMI. However, these authors did not observe significant changes in adiponectin concentrations after infliximab infusion, being the adiponectin preinfusion strongly correlated with adiponectin postinfusion. Two independent studies, the first performed by Nagashima et al and the second by Lewicki et al, observed in RA patients treated with anti-TNF agents that adiponectin levels increased significantly with the treatment [42, 43].

While we observed that HDL-C levels increased significantly in the ETA + MTX group at week 4 of treatment and this increase remained until the end of month 6, the HDL-C levels of patients who received MTX as monotherapy did not increase significantly during followup. This response of ETA in increasing HDL-C levels was also observed by Gonzalez-Juanatey et al. [27] in patients with RA, psoriatic arthritis, or ankylosing spondylitis who were treated with ETA, but not in patients treated with infliximab, attributing these findings to possible differences in the mechanism of action of ETA on lymphotoxin-α. Our findings support their observations, although we exclusively evaluated patients with RA. In other studies, Seriolo et al. observed an increase in HDL-C levels following the use of anti-TNF-α drugs for RA [31–33], but they did not make explicit whether this increase was observed only in patients receiving ETA or if it was also observed in patients treated with other anti-TNF agents included in their study (infliximab or adalimumab). The intrinsic mechanism by which ETA may increase levels of HDL-C is not well understood, although it may involve ETA blocking the expression of lymphotoxin-α. Lymphotoxin-α has been observed to possess proatherogenic properties in animal models. Absence of lymphotoxin-α is associated with a decrease in total cholesterol levels and an increase in HDL-C levels [12].

We failed to detect a correlation between baseline TNF-α serum levels and lipid profile, and we also observed an unexpected dissociation between modifications in HDL-C levels and changes in TNF-α levels; these findings suggest that other mechanisms independent of TNF-α blocking may contribute to the beneficial effects obtained with ETA + MTX on HDL-C levels. Other mechanisms that may affect dyslipidemia in patients with RA include genetic and environmental factors. Among genetic factors, several polymorphisms have been reported to be associated with dyslipidemia and cardiovascular diseases in RA. Vallvé et al. observed, in patients with RA, an association between TNF-α-1031 T/C genetic polymorphisms and LDL particles with greater susceptibility to oxidation [44]. Park et al. observed that the APOM C-1065A polymorphism is associated with higher risk for developing dyslipidemia in RA, because reduced HDL cholesterol levels were influenced by the APOM genotype [45]. Rodríguez-Rodríguez et al. observed an association between the TNFA rs1800629 (G > A) gene polymorphism and predisposition to cardiovascular complications in RA, including ischemic heart disease, cerebrovascular accidents, heart failure, and peripheral arteriopathy [46]. In another study, López-Mejías et al. identified an association between cardiovascular disease and the NFKB1-94ATTG ins/del (rs28362491) gene polymorphism [47].

Limitations of the present study include some differences at baseline that may influence the response observed in HDL-C, including age differences and a trend toward longer disease duration in the group receiving ETA. Because the present study is a cohort, there was no randomized selection of patients being included in one or the other treatment arm, increasing the possibility of variables that may confound the outcomes. Therefore, we cannot totally exclude other potential confounding factors that may exert an influence on the increase in HDL-C levels obtained with ETA + MTX treatment. However, we restricted the inclusion of relevant confounders associated with changes in lipid levels, such as administration of antimalarials, prednisone, or a multiplicity of NSAID drugs during the study period. However, for ethical reasons, we advised all patients to stop smoking and to increase exercise, which may have contributed to the observed effects on lipids levels (although it is relevant to consider that although both groups received this advice, only the ETA + MTX group improved significantly in HDL-C measurements).

In summary, we observed that combined treatment with ETA + MTX increased HDL-C levels of patients with RA, without significantly affecting other lipids. These findings were independent of changes in TNF-α levels and may represent new information with respect to a protective effect of ETA + MTX combination against the development of atherosclerotic complications in RA. Further studies should evaluate whether this effect has clinical relevance for the decrease of atherosclerotic consequences such as stroke or myocardial infarction in patients with RA.

## Figures and Tables

**Table 1:** Comparison of baseline characteristics between the etanercept + methotrexate (ETA + MTX) group and the MTX as monotherapy group.

Clinical characteristics	Total *n* = 35	ETA + MTX *n* = 22	MTX *n* = 13	*P*
Females, *n* (%)	32 (91)	20 (91)	12 (92)	0.88
Age, years	44.9 ± 9.7	47.4 ± 8.3	40.6 ± 10.8	0.04
Body mass index, kg/m^2^	25.0 ± 3.3	24.7 ± 3.4	25.5 ± 3.2	0.49
Smoking, *n* (%)	12 (34)	7 (32)	5 (39)	0.68
Duration of RA, years	10.8 ± 6.6	12.3 ± 6.7	8.2 ± 6.0	0.08
DAS-28 score	6.3 ± 0.9	6.2 ± 0.8	6.5 ± 1.1	0.47
HAQ-DI score	1.45 ± 0.56	1.45 ± 0.52	1.51 ± 0.64	0.78
Morning stiffness, mm (VAS)	62 ± 20	63 ± 19	61 ± 23	0.85
Severity of pain, mm (VAS)	67 ± 19	66 ± 16	69 ± 24	0.65
Disease activity, by physician, mm (VAS)	61 ± 16	61 ± 15	60 ± 17	0.91
Total cholesterol, mg/dL	181.5 ± 40.2	187.9 ± 38.1	170.6 ± 42.7	0.22
High total cholesterol levels, *n* (%)	12 (34)	7 (32)	5 (38)	0.73
Triglycerides, mg/dL	141.8 ± 67.4	150.4 ± 75.1	127.4 ± 51.5	0.34
High triglycerides levels, *n* (%)	14 (40)	9 (41)	5 (38)	0.89
HDL-C, mg/dL	46.7 ± 13.0	48.1 ± 15.6	44.3 ± 6.8	0.34
Low HDL-C levels, *n* (%)	8 (23)	6 (27)	2 (15)	0.68
LDL-C, mg/dL	100.7 ± 30.9	100.7 ± 25.8	100.5 ± 39.2	0.99
High LDL-C levels, *n* (%)	7 (20)	3 (14)	4 (31)	0.38
Dyslipidemia, *n* (%)	20 (57)	13 (59)	7 (54)	0.76
TNF-*α*, pg/mL	96.91 ± 169.26	129.14 ± 207.70	42.36 ± 23.46	0.08
Rheumatoid factor, U/mL	421.23 ± 633.92	501.59 ± 752.79	309.97 ± 422.72	0.42
C-reactive protein, mg/L	29.97 ± 46.31	20.08 ± 30.02	43.67 ± 61.11	0.17

HDL-C: high-density lipoprotein cholesterol; LDL-C: low-density lipoprotein cholesterol; TNF-*α*: tumor necrosis factor-alpha; VAS: visual analogue scale; RA: rheumatoid arthritis; DAS-28: disease activity score; HAQ-DI: Health Assessment Quest-Disability Index. Quantitative variables are presented as mean ± standard deviation (SD); qualitative variables are presented in number (%); comparisons between proportions utilized Fisher exact test; comparisons between means were calculated with unpaired Student's *t*-tests.

**Table 2:** Correlations (*r*) between baseline tumor necrosis factor-alpha (TNF-*α*) serum levels and selected variables.

Characteristic	TNF-α (pg/mL) *n* = 35	
	*r*	*P*
Age	−0.041	0.81
Disease duration	0.058	0.74
Joint tenderness count	0.079	0.65
Joint swelling count	0.035	0.84
DAS-28 score	0.061	0.72
HAQ-DI score	0.247	0.15
Disease activity (physician)	0.304	0.07
Total cholesterol	0.031	0.86
Triglycerides	−0.131	0.45
HDL-C	−0.114	0.51
LDL-C	−0.227	0.19
Rheumatoid factor	0.255	0.16
C-reactive protein	−0.089	0.63

HDL-C: high-density lipoprotein cholesterol; LDL-C: low density lipoprotein cholesterol; DAS-28: disease activity score; HAQ-DI: Health Assessment Quest-Disability Index; disease activity was assessed by the physician using a visual analogue scale (VAS) from 0 to 100 mm. Joint swelling or tenderness counts were taken from 28 joints. Correlations were computed with Pearson correlation test.

**Table 3:** Within-group comparisons over time of changes in selected clinical variables, C-reactive protein levels (CRP), and rheumatoid factor titers.

Characteristic	ETA + MTX			MTX		
	Baseline (*n* = 22)	4 weeks (*n* = 22)	24 weeks (*n* = 22)	Baseline (*n* = 13)	4 weeks (*n* = 13)	24 weeks (*n* = 13)
DAS-28 score	6.2 ± 0.8	3.8 ± 1.6^‡^	3.6 ± 1.4^‡^	6.5 ± 1.1	5.5 ± 1.3∗∗	4.9 ± 1.2^‡^
HAQ-DI score	1.45 ± 0.52	0.57 ± 0.43^‡^	0.57 ± 0.50^‡^	1.51 ± 0.64	1.13 ± 0.61^‡^	0.86 ± 0.47^‡^
Disease activity (physician)	61 ± 15	29 ± 17^‡^	25 ± 16^‡^	60 ± 17	43 ± 24^†^	41 ± 17^†^
Rheumatoid factor, U/mL	501.59 ± 752.79	458.77 ± 669.78	1386.57 ± 4465.90	309.97 ± 422.72	138.95 ± 186.08	84.68 ± 172.53∗
CRP, mg/L	20.08 ± 30.02	14.82 ± 17.72	41.58 ± 91.04	43.67 ± 61.11	23.19 ± 26.89	28.92 ± 43.14

ETA + MTX: etanercept plus methotrexate group; MTX: methotrexate as monotherapy group; DAS-28: disease activity score; HAQ-DI: Health Assessment Quest-Disability Index. Quantitative variables are expressed as mean ± standard deviation (SD). Intragroup comparisons were performed with the Student's *t*-test. ∗*P* = 0.03, ∗∗*P* = 0.01, ^†^ *P* = 0.001, and ^‡^ *P* < 0.001.

**Table 4:** Within- and between-group comparisons of relative changes (%) in lipid profile and tumor necrosis factor-alpha (TNF-*α*) serum level at 4 and 24 weeks.

Characteristics	ETA + MTX (*n* = 22)			MTX (*n* = 13)			Comparison between groups (Δ relative change)
	Mean ± SD	Absolute change	Relative change (%)	Mean ± SD	Absolute change	Relative change (%)	*P*
Total cholesterol, mg/dL							
Baseline	187.9 ± 38.1	—	—	170.6 ± 42.7	—	—	0.22
4 weeks	178.5 ± 38.6	−9.4	5.0	172.8 ± 32.7	+2.2	1.3	0.18
24 weeks	182.7 ± 44.8	−5.2	2.8	188.9 ± 33.0	+18.3	10.7	0.04
Triglycerides, mg/dL							
Baseline	150.4 ± 75.1	—	—	127.4 ± 51.6	—	—	0.34
4 weeks	141.8 ± 57.4	−8.5	5.7	129.3 ± 51.9	+1.9	1.5	0.96
24 weeks	151.3 ± 61.2	+0.9	0.6	151.8 ± 74.2	+24.5	19.2	0.62
HDL-C, mg/dL							
Baseline	48.1 ± 15.6	—	—	44.4 ± 6.9	—	—	0.34
4 weeks	52.0 ± 15.8∗∗	+3.9	8.2	45.5 ± 9.8	+1.2	2.6	0.22
24 weeks	57.1 ± 13.9^†^	+9.0	18.7	47.1 ± 9.7	+2.7	6.1	0.04
LDL-C, mg/dL							
Baseline	100.7 ± 25.9	—	—	100.5 ± 39.2	—	—	0.99
4 weeks	99.7 ± 30.0	−1.0	1.0	101.1 ± 31.6	+0.6	0.6	0.40
24 weeks	97.3 ± 44.7	−3.4	3.4	109.6 ± 27.9	+9.0	9.0	0.24
TNF-*α*, pg/mL							
Baseline	129.14 ± 207.7	—	—	42.36 ± 23.5	—	—	0.88
4 weeks	219.7 ± 228.6∗	+90.6	70.2	33.9 ± 12.9	−8.3	19.8	0.02
24 weeks	337.4 ± 205.9^‡^	+208.2	161.2	50.7 ± 24.1	+8.2	19.6	0.01

ETA + MTX: etanercept plus methotrexate; MTX: methotrexate as monotherapy; HDL-C: high-density lipoprotein cholesterol; LDL-C: low-density lipoprotein cholesterol; SD: standard deviation. Absolute change is the difference at 4 or 24 weeks versus baseline; relative change is the percentage change at 4 or 24 weeks versus baseline values. Δ: relative change between groups is a comparison of the differences in relative change (%) at 4 and 24 weeks between groups. The *P* values for the Δ calculations were obtained with unpaired Student's *t* tests. Intragroup comparisons between 4 and 24 weeks versus baseline were calculated with paired Student's *t*-tests.

∗ *P* = 0.03, ∗∗*P* = 0.02, ^†^ *P* = 0.009, and ^‡^ *P* = 0.007.
